# Requirements for Physician Competencies in Allergy: Key Clinical Competencies Appropriate for the Care of Patients With Allergic or Immunologic Diseases *A Position Statement of the World Allergy Organization*

**DOI:** 10.1097/WOX.0b013e3181651689

**Published:** 2008-02-15

**Authors:** Michael A Kaliner, Sergio Del Giacco, Carlos D Crisci, Anthony J Frew, Guanghui Liu, Jorge Maspero, Hee-Bom Moon, Takemasa Nakagawa, Paul C Potter, Lanny J Rosenwasser, Anand B Singh, Erkka Valovirta, Paul Van Cauwenberge, John O Warner, WAO Specialty and Training Council

**Affiliations:** 1 With special recognition of the contribution of Karen Henley, staff liaison to Council

## 

Allergic diseases are quite prevalent worldwide, and the incidence of allergy is increasing everywhere [1-7]. Because allergic and immunologic processes overlap all organ systems, allergy is not always taught in medical schools as a separate subject. Indeed, lack of recognition of the specialty and of the need to teach about allergic and immunologic diseases results in allergy not being included at all in some medical school curricula [8]. With an estimated 22% of the global population experiencing allergic and immunologic diseases, it is time to recognize and strengthen education in allergy and immunology [8].

The World Allergy Organization (WAO), an alliance of 74 national and regional allergy societies, created this consensus document to establish educational guidelines for worldwide application to help identify and correct allergy education and training deficiencies and to define appropriate competencies. In creating this consensus, it is recognized that each country has its own principles and goals in medical education at the undergraduate and postgraduate levels. This document defines what WAO considers medical practitioners should know to care appropriately for allergic patients.

## Background

Diseases with an allergic etiology can affect many organ systems and occur in response to a wide variety of environmental factors. Allergic diseases are among the most common causes of chronic medical problems in both adults and children and are associated with high morbidity. They carry a large socioeconomic burden [9-12] and can result in catastrophic anaphylaxis or fatal asthma attacks. Systemic hypersensitivity diseases include, among others, asthma, rhinoconjunctivitis, otitis, rhinosinusitis, urticaria, angioe-dema, eczema, food allergy, drug allergy, insect allergy, occupational allergic diseases, and anaphylaxis. Conventionally, allergic diseases have been divided into those associated with immunoglobulin E (IgE)-mediated hypersensitivity and those involving other forms of hypersensitivity [13]. As a medical specialty based on immunology, the allergy specialty (in some countries, called allergology) is concerned with prevention and diagnosis of the disease and management and rehabilitation of patients with allergic and related diseases.

In some countries, the allergy specialty is combined with clinical immunology. Immune processes are fundamental to host defense. Malfunction of the immune system causes infections, reduces immune surveillance, leads to autoimmune phenomena, and impacts every organ system. Clinical immunology relates to immune system dysfunctions and immunologically mediated diseases, which by definition also include allergic diseases. In some other countries, allergy is positioned as a component of organ-specific specialties such as dermatology, pulmonology, rheumatology, gastroenterology, and otorhinolaryngology; this positioning results in the specialty of allergy not always being recognized separately, and there may be no defined standardization of specialty training requirements for allergy. The WAO, as a global society, proposes that the best way to achieve a uniform quality level of care for the many millions of patients with allergic diseases is to define the key levels of competence required for both primary care clinicians and specialists who see patients who have allergic disorders.

Given the very high prevalence of allergic diseases and the different medical systems throughout the world, patients may be managed by primary care physicians, internists or pediatricians (who in this document are grouped and defined as first-level care), by organ-based specialists who receive some specific training in allergy and/or immunology (defined in this document as second-level care), and/or by fully trained specialists in allergy (third-level care). The WAO believes that an acceptable level of competence is required for all physicians who see allergy patients but who are not allergy specialists.

A strong cooperative network with vertical links among physicians and other health care professionals at the first-level of care, organ-based specialists, and allergists is necessary for the optimal management of allergy patients [14,15]. Which physician sees which patient and to whom the patient is referred reflects both the availability of physicians specifically trained in allergy and immunology and the levels of competence of the referring physicians. It is essential for proper medical management that first- and second-level physicians are cognizant of the importance of an accurate diagnosis and the appropriate point at which to refer a patient to the next level of care.

This document recommends the appropriate levels of competence necessary to manage allergic patients at each of the 3 defined levels and clarifies the appropriate time point in the disease for referral to an allergist. Once agreement upon these recommendations is achieved, the WAO will develop a more specific core curriculum and appropriate educational and training programs for medical students, residents in training, general practitioners, pediatricians, internists, organ-based specialists, and allergy specialists.

It is proposed that the levels of competence for knowledge and skills be divided as described in the following paragraphs.

### First-Level Care

This level includes recommendations for the knowledge and skills in allergy required for general practitioners, internists, and pediatricians. It also includes the knowledge and skills recommended for family practitioners and specialists in regions where organ-based specialists are not formally trained in the allergic aspects of their specialty and where trained allergists are not available. These recommendations also will apply to nurse practitioners and physicians' assistants if they are part of the health care community.

Knowledge at this level should include a background in immunology obtained during medical training and should include an understanding of hypersensitivity mechanisms (Gell and Coombs I-IV); major mechanisms of host defense; the role of immunoglobulins in host defense; knowledge of lymphocyte function; the roles of leukocytes, especially eosinophils; and the functions of mast cells and basophils.

Knowledge at the first level of care should include the following areas:

1. Adequate clinical knowledge about the main allergic diseases, including rhinoconjunctivitis, rhinosinusitis, otitis, asthma, urticaria, angioedema, eczema, food allergy, insect allergy, anaphylaxis, drug allergy, and immunodeficiency, so that the diagnosis and treatment of both acute and chronic diseases are possible. Where feasible, such care should be carried out in collaboration with or with access to an allergist or an allergy referral center.

2. Adequate knowledge in the interpretation of the main diagnostic allergy tests, skin prick tests, and serological tests for IgE and an understanding of pulmonary function test interpretation. Such training generally would not include competency in performing skin tests or the more sophisticated pulmonary function tests.

3. Sufficient training to recognize patients with a level of persistence or severity, who experience exacerbations that are life affecting or who have difficult-to-manage allergic or immunologic disease who should be referred to an allergy specialist for evaluation and initiation of treatment before the disease advances to a severe or life-threatening stage.

4. Immunotherapy (injective, sublingual) is performed by first-level physicians and other health care professionals in some countries. The WAO suggests that this is only appropriate as follows:

A. The immunotherapy has been prescribed by a specialist.

B. The first-level physician and other professionals have had adequate training in allergy and the recognition and management of anaphylaxis to provide this service safely.

C. The location where immunotherapy is performed fulfills all the conditions for patient safety. The site where immunotherapy is performed should be equipped at a minimum with:

a. oxygen and appropriate oxygen administration apparatus (mask or nasal device) and a manual or automatic respiration device;

b. epinephrine to be administered intramuscularly or intravenously;

c. antihistamines (both H1 and H2) for injection;

d. corticosteroids;

e. vasopressors;

f. colloid plasma expanders;

g. tourniquet to be placed above the site of injection of the vaccine; and

h. there must be good access to full resuscitation facilities within the building or at a nearby hospital resuscitation unit.

It is recommended that immunotherapy be initiated by an allergist or in a referral center and that a suitably trained first-level physician should provide maintenance treatment only.

### Second-Level Care

Recommendations for key competencies at the second level of care apply to organ-based physicians such as those in dermatology, pulmonology, gastroenterology, otorhinolaryngology, and rheumatology, who see allergy patients or act as allergy specialists, receiving referrals of allergy patients for diagnosis and management. In some health care systems, second-level care physicians receive training specifically in allergy. Knowledge at this level should include a fundamental background in allergy and immunology, an understanding of common allergic diseases, and the knowledge and skills to perform and interpret diagnostic tests to competently treat uncomplicated allergic diseases.

In most countries, background training in allergy and immunology is obtained through rotations in allergy and immunology centers provided during residency in internal medicine or pediatrics. Thereafter, during the 2 to 3 years of training in specialties such as dermatology, pulmonology, otorhinolaryngology, gastroenterology, or rheumatology, adequate opportunities for instruction in allergy and immunology should be required. Organ-based specialists at this level should be required to have the knowledge base required of any first-level primary care physician, plus additional knowledge of host defense and clinical immunology and some understanding of cytokines and chemokines, genetics and environmental factors, and allergens and their relationship to human diseases [16,17].

The recommendations for second-level organ-based specialists include the following:

1. Broad clinical knowledge of major allergic and immune deficiency diseases.

2. Knowledge sufficient to diagnose and treat the common uncomplicated cases of allergic disorders according to national and international guidelines.

3. Adequate skills to perform and interpret allergy skin tests and the ability to interpret the other tests useful for the diagnosis, treatment, and prevention of allergic diseases.

4. Administration of various forms of immunotherapy (in collaboration with allergy specialists and referral centers) after adequate training, but only if such therapy is performed in a setting where patient safety is ensured (see First-Level Care, 4.C.)

### Third-Level Care

Third-level specialists are fully trained in allergy, having spent 2 or 3 years in training beyond either internal medicine or pediatric training. Such specialists will be expected to have a full knowledge and complete set of skills relating to all allergic diseases, and the competence to diagnose and treat in all areas.

A core training needs to be acquired before the specific training in allergy. This training will be in internal medicine for those intending to deal with adult allergy patients, and in pediatrics for those intending to deal with children. In certain countries (the United States, for instance), training in allergy encompasses both pediatrics and internal medicine.

## Knowledge Objectives

1. Immune mechanisms involved in the development of immunologically mediated diseases and, in particular, allergic sensitization and disease formation.

2. Genetic and environmental factors, including infectious diseases, involved in the genesis of allergic diseases.

3. Pathogenesis of rhinoconjunctivitis, otitis, rhinosinusitis, asthma, atopic dermatitis, urticaria, and angioedema; drug and food allergy; insect allergy and anaphylaxis; and the concept that many allergic diseases are systemic in etiology.

4. Relationship between tissue inflammation and repair.

5. Mechanisms of IgE-mediated immediate- and late-phase allergic reactions.

6. Mechanisms of non-IgE-mediated allergic reactions and other disorders in the differential diagnosis of allergic disease. These diseases include, but are not limited to, nonallergic rhinitis; drug-induced rhinitis; acute and chronic rhinosinusitis; nonallergic asthma; cough; bronchitis; non-IgE-mediated anaphylaxis; idiopathic urticaria; eczema; otitis; conjunctivitis; eosinophilic esophagitis, gastroenteritis, and colitis; celiaclike syndromes; food-induced enteropathies leading to gastroesophageal reflux, esophagitis, gastritis, and gut motility disorders including constipation.

7. National and global epidemiology of allergic diseases.

8. Local airborne, contact, and occupational allergens.

9. Classification and relative importance of all relevant allergens and their biologic characteristics, including heat, digestive stability, and cross-reactivity; understanding of local pollen counts and the characteristics of various aeroallergens and routes of allergen exposure.

10. Therapy.

A. Use and route of administration of antihistamines; mast cell stabilizers; bronchodilators; nasal, oral, topical, and inhaled glucocorticosteroids; decongestants; leukotriene modifiers; theophylline; adrenergic agonists; anticholinergics; mucolytics; antibiotics; adrenaline; and all other pharmacological and immunologic agents used to treat allergic and immunologic diseases.

B. Use of emollients, antibiotics, topical glucocorticosteroids, immune modulators, and all other agents and techniques used to manage eczema and other allergic skin disorders.

C. Use of immune modulators, such as specific allergen immunotherapy, monoclonal antibodies, including anti-IgE, and immunoglobulin replacement used to treat allergic and immunologic disorders, and knowledge of immune modulators that are being developed for clinical use in allergic and immunologic disorders.

D. Methods and value of allergen-avoidance techniques.

E. Avoidance diets and nutritional implications of dietary modification.

F. Knowledge of national and international guidelines for the management of allergic and immunologic disorders in adults and children, with particular emphasis on safety and efficacy of all therapies.

11. Investigation and management of adverse reactions to drugs and vaccines.

12. Methods to measure cells and mediators in biologic fluids and tissues.

13. Primary and secondary prevention of allergy, particularly in children.

14. Understanding of the social and psychological issues associated with allergic diseases.

15. Diagnosis and management of occupational allergic diseases.

16. Methods to monitor home or work environments for allergens associated with allergic diseases.

17. Understanding of environmental factors such as pollutants and occupational allergens and of viral respiratory tract infections that affect allergic sensitization and disease development.

18. Recognition/diagnosis and treatment or appropriate referral of patients with humoral and cellular immunodeficiencies, hereditary and acquired complement deficiencies, and phagocytic disorders, depending upon whether the specialist is an allergist or allergist/clinical immunologist.

## Skills Objectives

1. Clinical skills.

A. Differential diagnosis, evaluation, and management or appropriate immunologic referral of the following:

a. Eczema

b. Rhinoconjunctivitis

c. Conjunctivitis

d. Rhinosinusitis

e. Atopic dermatitis

f. Asthma, cough, dyspnea, and recurrent wheeze

g. Acute and chronic urticaria, including physical urticarias

h. Angioedema, including hereditary angioedema

i. Anaphylaxis

j. Food allergy and intolerance

k. Drug and vaccine allergies or intolerance

l. Insect allergy/hypersensitivity

m. Oral allergy syndrome

n. Latex allergy

o. Occupational allergy, asthma, eczema

p. Otitis

q. Common variable immunoglobulin deficiency and related immunodeficiencies

r. Primary immunodeficiencies

s. Secondary immunodeficiencies

t. Complement deficiencies

u. Abnormalities of phagocytic cells

2. Management of patients with multiple or complex allergies.

3. Management of patients with multiple food allergies, requiring avoidance diets.

4. Provision of allergen avoidance advice.

5. Safe supervision of food and drug challenges.

6. Assessment of patients for immunotherapy. Proper administration of immunotherapy including immunotherapy dose adjustment and management of complications. Supervision of immunotherapy protocols. Recognition and management of allergic reactions associated with immunotherapy.

7. Recognition of indications for and the skills to perform, interpret, and understand the limitations of skin prick, intradermal, patch, and delayed-type skin tests, and specific in vitro IgE antibody tests.

8. Interpretation of natural allergen and environmental exposures.

9. Evaluation and differentiation of non-IgE-mediated hypersensitivity reactions.

10. Investigation and management of behavioral problems related to allergic and immunologic diseases.

11. Improvement of patient compliance with pharmacotherapy regimens through personalized disease management plans.

12. Knowledge of drug desensitization protocols.

13. Management in the community of patients at risk of anaphylactic reactions, incorporating an understanding of integrated care pathways.

14. Diagnosis, treatment, and/or referral of primary and secondary humoral and cellular immunodeficiencies Such diseases include, but are not limited to Bruton agammaglobulinemia, severe combined immunodeficiency, thymic dysplasia, adenosine deaminase deficiency, Wiskott-Aldrich syndrome, ataxia telangiectasia, and various lymphocyte activation defects.

15. Safe and effective administration of intravenous gamma globulin.

16. Recognition and management or appropriate referral of hereditary and acquired complement deficiencies.

17. Knowledgeaboutand treatment/referralof phagocyticcell disorders, such as Chédiak-Higashi syndrome, chronic granulomatous disease, leukocyte adhesion defects, and a variety of congenital and acquired neutropenias.

## Technical Skills and Knowledge Objectives

1. Performance and interpretation of skin prick, intradermal, patch tests, and delayed hypersensitivity tests.

2. Performance of diagnostic testing for suspected drug, biologic, or vaccine allergy.

3. Safe preparation and administration of immunotherapy vaccines.

4. Performance of allergen provocation tests, such as nasal, conjunctival, bronchial, and oral challenges, and food and medication challenges.

5. Performance of patch testing for contact dermatitis.

6. Performance or knowledge of rhinoscopy and laryngoscopy, nasal endoscopy, acoustic rhinometry,^1 ^and rhinomanometry.^1^

7. Performance of basic lung function testing, including spirometry and bronchial provocation tests (methacholine or histamine challenges, measurement of flow-volume loops and pulse oximetry, and prebronchodilator and postbronchodilator testing).

8. Knowledge of how and when to measure exhaled nitric oxide, and how and when to perform whole-body plethysmography and impulse oscillometry.^1^

9. Knowledge of how and when to use various tests to measure airway inflammation and/or constriction, including bronchodilator-induced bronchodilation, induced sputum,^1 ^and/or bronchial and bronchoalveolar lavage.^1^

10. Assessment of environmental hazards in occupational allergy and knowledge of live insect sting challenges.

11. Management of exclusion diets and provocation diets.

12. Knowledge of and ability to interpret measurements of immune function, including serum immunoglobulin levels, IgG subclass levels, preimmunization and post-immunization antibody titers, isohemagglutinin titers, and other ancillary tests for use in the differential diagnosis of congenitalor acquired humoral immunodeficiency.

13. Measurement and interpretation of laboratory tests to diagnose hereditary angioedema and complement deficiencies.

14. Measurement of phagocytic function.

15. Interpretation of electrocardiograms, chest radiographs, computerized tomography scans and magnetic resonance images of the chest and sinuses, and interpretation of the main laboratory tests (blood, serum, microbiological, urine, and fecal tests).

## Attitudes

1. Ability to work with colleagues in other disciplines.

2. Appreciation of the scope and limitations of allergy testing.

3. Appreciation of the limitations and problems created by so-called complementary medicine or alternative allergy practices.

4. Understanding of the role of patient support groups and ability and willingness to work with patient support organizations.

5. Appreciation of all the issues relating to patient confidentiality and the ethical standards expected of all physicians.

6. Understanding of research protocols, the ethics of experimental design, data analysis, biostatistics, good clinical practice, and good laboratory practice, and a willingness to become involved in either clinical or basic translational research.

7. Knowledge of the country-specific legal framework for reporting of occupational diseases and assisting patients in obtaining compensation for occupational diseases.

8. An ability to be a clinical decision maker, communicator, collaborator, manager, health care advocate, and scholar.

## End Note

^1^Some of these skills should be at least taught and understood but may not be performed personally, in accordance with national guidelines and established practice parameters.
